# Thanks to our reviewers of 2016 and call to review in 2017 for us

**DOI:** 10.1080/13814788.2017.1291936

**Published:** 2017-02-24

**Authors:** Carl Llor, Manfred Maier, Marie-Eve Rougé Bugat, An de Sutter, Igor Švab, José Maria Valderas, Henk van Weert, Adam Windak, Jelle Stoffers

The Editors of the *European Journal of General Practice* should like to express their gratitude to all reviewers who have advised us during the year 2016. To acknowledge their indispensable contribution to the *European Journal of General Practice*, we have listed the names of all referees who completed at least one review report between 16 December 2015 and 16 December 2016.

Heinz-Harald Abholz, Erik Abilsnes, Jeffery Adams, Gisele Apter, Enric Aragonés, Chris Arden, Davut Baltaci, Tadej Battelino, Carol Bayona, Jaume Benavent, Ria Benko, Anette Berendsen, Christophe Berkhout, Sonja Bidmon, Peter Birner, Bjørn Bjorvatn, Marco Blanker, Jan Bosmans, Colin Bradley, Carlos Brotonos, Mateja Bulc, L.H. Burridge, Manuel Campiñez, Meltem Ciceklioglu, Peter Clarys, David Cohen, Gloria Cordoba, Lizzie Cottrell, Anne-Marie Cox, An de Sutter, Jan Degryse, Dorner, Thomas, Chris Dowrick, Sandra Fahrenkrog, Gabriele Fischer, Thomas Frese, Zbigniew Gaciong, Luis Galvez, Jochen Gensichen, Sherly George, Juan Gervas, Christoph Gisinger, Liam Glynn, Maciek Godycki-Cwirko, Alexandre Gouveia, Tomasz Grodzicki, Dilek Güldal, Ronny Gunnarsson, Peter Hjertholm, Kathryn Hoffmann, Eva Hummers-Pradier, Thomas Kahan, Laszlo Kalabay, Ruth Kalda, Gustav Kamenski, Bridget Kane, Brian Kelly, Martina Kelly, Marko Kolšek, Evan Kontopantelis, Dionne S. Kringos, Ron Kusters, Ilias-Ioannis Kyriopoulos, Toine Lagro, Amnon Lahad, A. J. Larner, Asam Latif, Klaus Linde, Christos Lionis, Carl Llor, José Ramon Loayssa, Nicole Lowres, Heidi Lyshol, Roar Maagaard, Aldo Pietro Maggioni, Christian Mallen, Niina Markkula, Inaki Martin-Lesende, Mercè Marzo-Castillejo, Marion McAllister, Saskia Mol, Jose M. Molero, Ana Moragas-Moreno, Maria Luisa Morato, Tania Morris, Luke Mounce, Anders Munck, Miguel Muñoz, Peter Murchie, Markus Nielen, Caitlin Notley, Peter Nygaard, Abimbola Obimakinde, Zeliha Ocek, Eric Olsman, Graziano Onder, Ennio Ongini, John Paget, Davorina Petek, Soeren Prins, Enriqueta Pujol-Ribera, Jesus Pujol, Maja Racic, S. Ramlall, Sofia B. Ravara, Shmuel Reis, Claire Rondet, Jim Ross, Danica Rotar Pavlič, Marie-Eve Rougé Bugat, Guy Rutten, Antonio Sarría Santamera, Willemijn Schäfer, Nynke Scherpbier, Henrik Schroll, Michaela Schunk, Polona Selic, Monika Seltenhammer, Helmut Sinzinger, Jef Sisler, Antoni Sisó Almirall, Marleen Smits, Hilde Solberg, Sal Stapley, Igor Ŝvab, Dorota Szczesniak, Angela Taft, Clare Taylor, Gabriela Heiden Telo, Berend Terluin, Bratt Thombs, Tomasz Tomasik, Salvador Tranche, Michael J. Twigg, Anne-Marie Uijen, Steven Uittenbogaart, Sabina Ulbricht, Aysegul Uludag, Juan Valdes-Stauber, Joan Antoni Valles, Wil van den Bosch, Marc van der Wel, Tineke van Geel, Evelien van Riet, Piet Vanden Bussche, Juha Varis, José Vázquez Villegas, Theo Verheij, Ernest Vinyoles, Johannes Wancata, Jane Wilcock, Oriol Yuguero Torres.

We want to thank you all for your appreciated assistance! Your review reports were very valuable feedback to the authors and helped us to make our editorial decisions. Currently, the *European Journal of General Practice* accepts around 30% of all submitted manuscripts.

Although our database of authors and reviewers steadily increases, the Editors do not always find it easy to find referees within the journal’s deadlines. Therefore, we sincerely hope that all reviewers want to continue their review work in the future. If you would like to update your reviewer account, e.g. to specify your expertise, please visit http://mc.manuscript-central.com/ejgp and log in to modify your profile. In addition, we invite all readers of the *European Journal of General Practice* who would like to review manuscripts for this journal, to visit http://mc.manuscriptcentral.com/ejgp and to register as ‘new user’, i.e. to complete the details of their expertise. We provide a format to facilitate the review.

If you have comments regarding the journal or its peer review process, we invite you to contact the editorial office (Ms. Anneke Germeraad-Uriot, Editorial Assistant: ejgp-agermeraad@maastrichtuniversity.nl).

